# MicroRNA-340-5p suppresses non-small cell lung cancer cell growth and metastasis by targeting ZNF503

**DOI:** 10.1186/s11658-019-0161-1

**Published:** 2019-05-28

**Authors:** Guojie Lu, Yaosen Zhang

**Affiliations:** Department of Thoracic Surgery, Guangzhou Panyu District Central Hospital, No. 8, Fuyu East Road, Qiaonan Street, Panyu District, Guangzhou, 511486 People’s Republic of China

**Keywords:** Cell proliferation, Metastasis, miR-340-5p, NSCLC, ZNF503

## Abstract

**Background:**

MicroRNAs (miRNAs) have been reported to play crucial roles in cancer cell processes, including proliferation, metastasis and cell cycle progression. We aimed to identify miRNAs that could act as suppressors of cell growth and invasion in non-small cell lung cancer (NSCLC).

**Methods:**

Fifteen paired NSCLC tissue samples and pericarcinomatous normal tissues were collected and preserved in liquid nitrogen. The expression levels of miR-340-5p and ZNF503 mRNA were detected using a qPCR assay. The transfection of plasmids was conducted using Lipofectamine 3000 according to the manufacturer’s protocol. Cell proliferation was determined using a CCK-8 assay. The protein levels of endothelial–mesenchymal transition markers were measured using a western blot assay. Cell invasive ability was evaluated using a transwell assay. TargetScan was used to predict targets of miR-340. A dual luciferase reporter assay was performed to confirm a potential direct interaction between miR-340-5p and ZNF503.

**Results:**

The expression level of miR-340-5p was frequently found to be lower in NSCLC tissues than in matched pericarcinomatous normal tissues. Overexpression of miR-340-5p significantly inhibited the proliferation and invasion NCI-H1650 (a NSCLC cell line), while inhibition of miR-340-5p stimulated cell growth. Using TargetScan, we predicted that ZNF503 could be a target of miR-340-5p. Further mechanistic studies demonstrated that the forced expression of ZNF503 could partially abrogate the miR-340-5p-mediated decrease in NCI-H1650 cell viability and invasion, suggesting that miR-340-5p suppressed cell growth and invasion in a ZNF503-dependent manner.

**Conclusion:**

Our findings indicate that miR-340-5p inhibits NCI-H1650 cell proliferation and invasion by directly targeting ZNF503 and that miR-340-5p can serve as a potential therapeutic target for treating NSCLC.

**Electronic supplementary material:**

The online version of this article (10.1186/s11658-019-0161-1) contains supplementary material, which is available to authorized users.

## Introduction

Lung cancer, which is the most malignant form of cancer, exhibits the fastest growth in morbidity and mortality worldwide [1]. Based on histological subtypes, it is divided into non-small cell lung cancer (NSCLC) and small cell lung cancer (SCLC), with NSCLC accounting for 85% of cases [2]. Although therapeutic strategies have advanced over the past two decades, only 11% of patients experience an overall survival rate of 5 years [3]. Tumor metastasis contributes to the high mortality, implying that more effective targeted therapies are required to improve the overall survival [4, 5]. Searching for the genes that drive cancer metastasis and targeting them may be a practical approach for developing an effective treatment for NSCLC.

MicroRNAs (miRNAs) are non-coding RNAs that regulate gene expression by binding to the 3′-untranslated region (UTR) of mRNA [6]. A considerable number of studies have shown that miRNAs are involved in the development and progression of various tumors [7]. For instance, miR-10b, an onco-miR, facilitates metastasis in breast cancer and glioblastoma [8, 9]. MiR-155 also plays an oncogenic role in many types of tumors, including lung cancer [7].

By contrast, some miRNAs possess a tumor suppressive function. It has been reported that members of the miR-200 family suppress metastasis and angiogenesis, and induce vascular normalization in lung cancer [10]. Trang et al. demonstrated that the systemic delivery of tumor-suppressing miR-34a mimics reduced the tumor area in mice with lung cancer [11].

Increasing evidence suggests that miR-340 also acts as a tumor suppressor. For example, miR-340 inhibits cell mobility and invasion by decreasing the mRNA level of MYO10 in breast cancer [12]. Huang et al. [13] found that miR-340 suppresses cell growth and metastasis by negatively regulating the MDM2 protein in prostate cancer. MiR-340-5p can enhance the sensitivity of osteosarcoma to cisplatin [14]. In NSCLC, a lower level of miR-340 expression correlates with a poor prognosis and increases cell viability because of miR-340 targeting CDK4 [15]. Fernandez et al. [16] have shown that miR-340 suppresses NSCLC cell growth and elevates cell death rates by controlling levels of p27 at both the translational and post-translational levels. However, the underlying mechanism of miR-340-5p inhibition of NSCLC metastasis remains poorly understood.

In this study, we found that miR-340-5p is downregulated in NSCLC tissues relative to its expression in pericarcinomatous normal tissues. Further mechanistic studies demonstrated that miR-340-5p inhibits NCI-H1650 cell proliferation and invasion by targeting the ZNF503 protein.

## Materials and methods

### NSCLC tissue specimens

Paired NSCLC tissue and pericarcinomatous normal tissue samples were collected from 15 NSCLC patients who were undergoing surgical resections without prior chemotherapy or radiotherapy in the Guangzhou Panyu District Central Hospital in 2017 and 2018. The samples were preserved in liquid nitrogen for further study. Before surgery, all patients signed an informed consent form. This project was approved by the Guangzhou Panyu District Central Hospital Institutional Review Board, and all procedures were in accordance with the principles of the Declaration of Helsinki.

### Cell culture

The normal lung cell line BEAS-2B and NSCLC cell lines A549, NCI-H460, NCI-H1299, NCI-H1650 and NCI-H292 were purchased from the Chinese Academy of Sciences Cell Bank. BEAS-2B was cultured in BEBM medium (Lonza/Clonetics Corporation) that was supplemented with 10% (v/v) fetal bovine serum (FBS; Thermo Fisher Scientific). The NSCLC cell lines were cultured in RPMI-1640 medium containing 10% FBS. Cells were maintained at 37 °C in a humidified incubator with 5% CO_2_.

### RNA extraction and real-time PCR

MiRNA from tissues and cells and total mRNA from cells were respectively extracted using the miRNeasy Mini Kit (Qiagen) and the RNAiso Plus Kit (Takara Bio). cDNA was synthesized using EasyScript One-Step gDNA Removal and cDNA Synthesis SuperMix (Transgen Biotech) and the reverse transcription of miRNA was performed using a miScript II RT Kit (Qiagen). qPCR was conducted on a CFX96 Real-Time Thermocycler (BioRad) using SsoAdvanced Universal SYBR Green Supermix (BioRad). U6 and GAPDH were regarded as internal controls for normalizing miR-340-5p and ZNF503, respectively. Relative mRNA expression was derived using the 2^-ΔΔCT^ method. The sequences [12] of all primers used in this assay are:

miR-340 forward, 5′-GCGGTTATAAAGCAATGAGA-3′;

miR-340 reverse, 5′-GTGCGTGTCGTGGAGTCG-3′;

U6 forward, 5′-GCTTCGGCAGCACATATACTAAAAT-3′;

U6 reverse, 5′-CGCTTCACGAATTTGCGTGTCAT-3′;

ZNF503 forward, 5′-CAAACTCTCCTCGGTTGCCT-3′;

ZNF503 reverse, 5′-GGGTTTGGAGTACGGCTTGA-3′;

GAPDH forward, 5′-TGCACCACCAACTGCTTAGC-3′;

GAPDH reverse, 5′-GGCATGGACTGTGGTCATGAG-3′.

### Transfection assay

NCI-H1650 cells were seeded in 6-well plates and cultured overnight. After logarithmic cell growth reached 80% confluence, the cells were transfected with the indicated plasmids using Lipofectamine 3000 (Thermo Fisher Scientific), according to the manufacturer’s protocol. MiR-340-5p mimics, inhibitors and scramble sequences were synthesized by GenePharma. pCMV6-ZNF503 was purchased from Origene Technologies Inc.

### Western blot assay

Total protein was extracted from NCI-H1650 cells using RIPA lysis buffer (Thermo Fisher Scientific). A BCA kit (Thermo Fisher Scientific) was used to detect the protein concentration in different samples. Proteins (40 μg) were separated using 10% SDS-PAGE and then blotted onto PVDF membranes (Millipore Sigma). Next, the PVDF membrane was blocked with 5% nonfat milk (BD Biosciences) at room temperature for 1 h and then incubated at 4 °C overnight with primary antibodies: E-cadherin (1:1000), vimentin (1:1000), β-actin (1:1000; all from Cell Signaling Technology) and ZNF503 (1:1000; from Abcam). Finally, the membrane was incubated with HRP-conjugated secondary antibodies (Cell Signaling Technology) at room temperature for 1 h, and enhanced chemiluminescence (Bio-Rad Clarity Western ECL) was used to visualize the protein bands.

### Cell viability assay

After transfecting with miR-340-5p mimics, inhibitors and pCMV6-ZNF503, the cells were plated in 96-well plates at a density of 1000 cells per 100 μl per well in a total volume of 200 μl. They were cultured overnight. Cell proliferation was measured using CCK-8 (Beyotime Biotechnology) at different times (0, 24, 48 and 72 h) using a Microplate Reader (Bio-Rad).

### Invasion assay

After transfection, 1 × 10^4^ cells/100 μl were resuspended in serum-free medium and seeded into transwell inserts (8 μm, Corning) that were coated with Matrigel (BD Biosciences). Complete medium (600 μl) was added to the bottom chamber and 48 h later the wells were fixed with 4% paraformaldehyde for 30 min and stained with 0.5% Crystal Violet for 30 min at room temperature. Cells in the upper chambers were removed with cotton swabs and those cells that had invaded to the lower side of the membrane were counted as the number of cells in three random fields under a microscope (Shinjuku).

### Luciferase reporter assay

Luciferase reporter vectors containing the seed sequence for miR-340 corresponding to the wild-type (WT) 3′-UTR of ZNF503 and a mutated version of the 3′-UTR of ZNF503 containing a mutation (Mut) within the core binding site for miR-340 were constructed. HEK293T cells were transiently co-transfected with WT-3′-UTR-ZNF503 or Mut-3′-UTR-ZNF503 and miR-340-5p mimics or the scrambled control using Lipofectamine 3000, according to the manufacturer’s protocol. Luciferase activity was measured 36 h after transfection using a Dual Luciferase Reporter Assay System (Promega Corporation). Renilla luciferase activity was used as the internal control, and data are expressed as the ratio of firefly to renilla luciferase activities.

### Annexin V/PI staining assay

Cells were transfected with different concentrations of miR-340-5p mimics (0, 2, 4, 6, 8, 10 nM) for 48 h. Next, cells were collected and stained with annexin V-FITC and propidium iodide (PI; BestBio). The fluorescence intensity of annexin V-FITC and PI was determined using a flow cytometer (Millipore). The apoptosis rate was analyzed with Flow Jo 10.0.7 software.

### Statistical analysis

Data were analyzed using GraphPad Prism 5.0 software and are presented as the means ± SD. The differences between two groups or among multiple groups were respectively calculated using an unpaired Student t-test or a one-way ANOVA. *p* < 0.05 was considered significantly different.

## Results

### MiR-340-5p is expressed at a low level in NSCLC tissues and cell lines

**Fig. 1:**
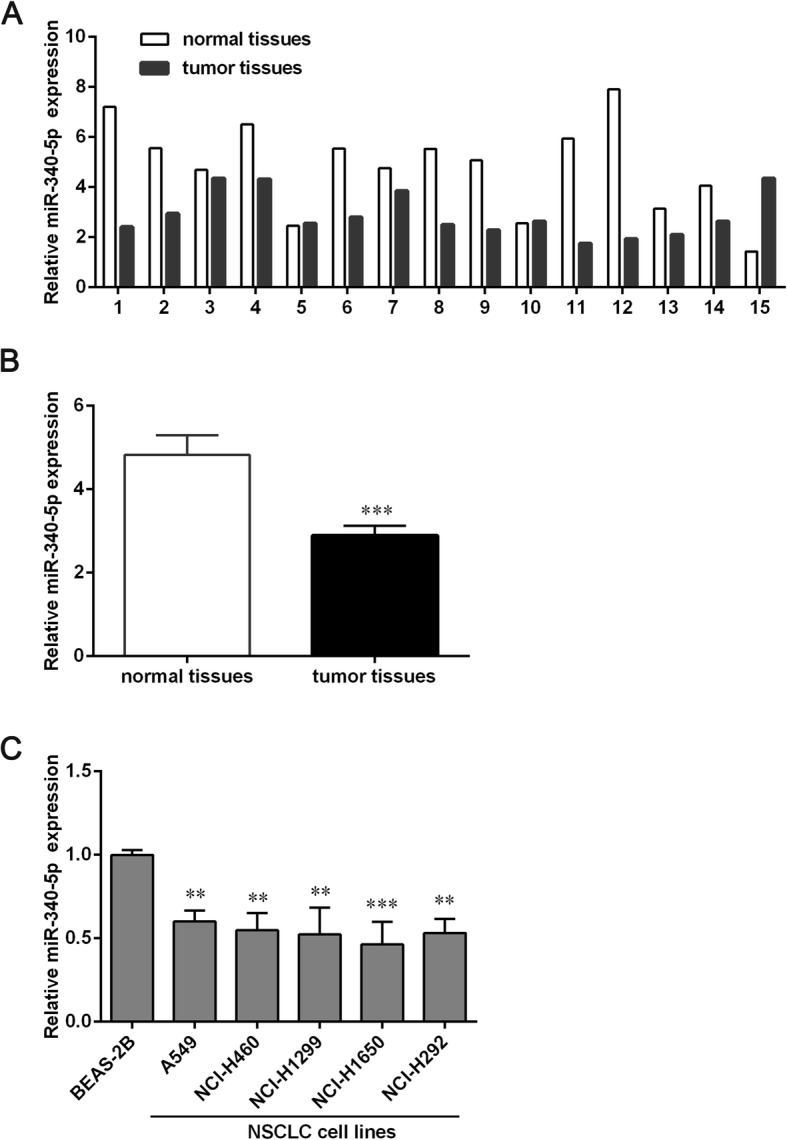
Expression of miR-340-5p in NSCLC tissues and cell lines. **a** The mRNA levels of miR-340-5p in 15 paired NSCLC tissue and pericancerous normal tissue samples were determined using qPCR. **b** The expression of miR-340-5p is significantly lower in NSCLC tissues than in normal lung tissues. **c** qPCR analysis of miR-340-5p expression in normal lung cells (BEAS-2B) and in five NSCLC cell lines (A549, NCI-H460, NCI-H1299, NCI-H1650 and NCI-H292) is shown (*n* = 3). Data are presented as the means ± SD. **p < 0.01 and ***p < 0.001 versus the normal tissues group or the BEAS-2B group

To explore the role of miR-340-5p in NSCLC, we determined the expression levels of miR-340-5p in paired NSCLC tissue and pericarcinomatous normal tissue samples from 15 patients. The results show that the expression of miR-340-5p was significantly lower in NSCLC tissues than in normal tissues, indicating that miR-340-5p may have an anti-tumor effect (Fig. 1a and b).

To determine the clinical significance of miR-340-5p in NSCLC, the relationship between miR-340-5p expression and clinicopathological parameters in the 15 NSCLC patients was analyzed. As shown in Additional file 4: Table S1, a high expression of miR-340-5p is significantly negatively related to advanced clinical stages (*p* = 0.0406) and tumor metastasis (*p* = 0.0406). Furthermore, we found that NSCLC cell lines, including A549, NCI-H460, NCI-H1299, NCI-H1650 and NCI-H292, had low levels of miR-340-5p compared to the normal lung cell line BEAS-2B. The lowest expression of miR-340-5p was in NCI-H1650. We chose this cell line for further study.

### Overexpression of miR-340-5p inhibits proliferation and invasion of NCI-H1650 cells

To clarify whether overexpression of miR-340-5p causes cell apoptosis, we conducted a screening experiment to investigate the effects of various concentrations of miR-340-5p mimics on apoptosis. Cells were transfected with different concentrations of miR-340-5p mimics (0, 2, 4, 6, 8, 10 nM) for 48 h, and then an annexin V/PI staining assay was applied to determine the apoptosis rate. As shown in Additional file 4: Table S2, the apoptosis rate induced by 0, 2 or 4 nM of miR-340-5p mimics was less than 10% while 6, 8, or 10 nM of miR-340-5p mimics let to an apoptotic rate above 10%. In addition, the overexpression effect of 4 nM miR-340-5p mimics was better than that of 2 nM miR-340-5p mimics. Thus, we chose 4 nM of miR-340-5p mimics for further study.

**Fig. 2:**
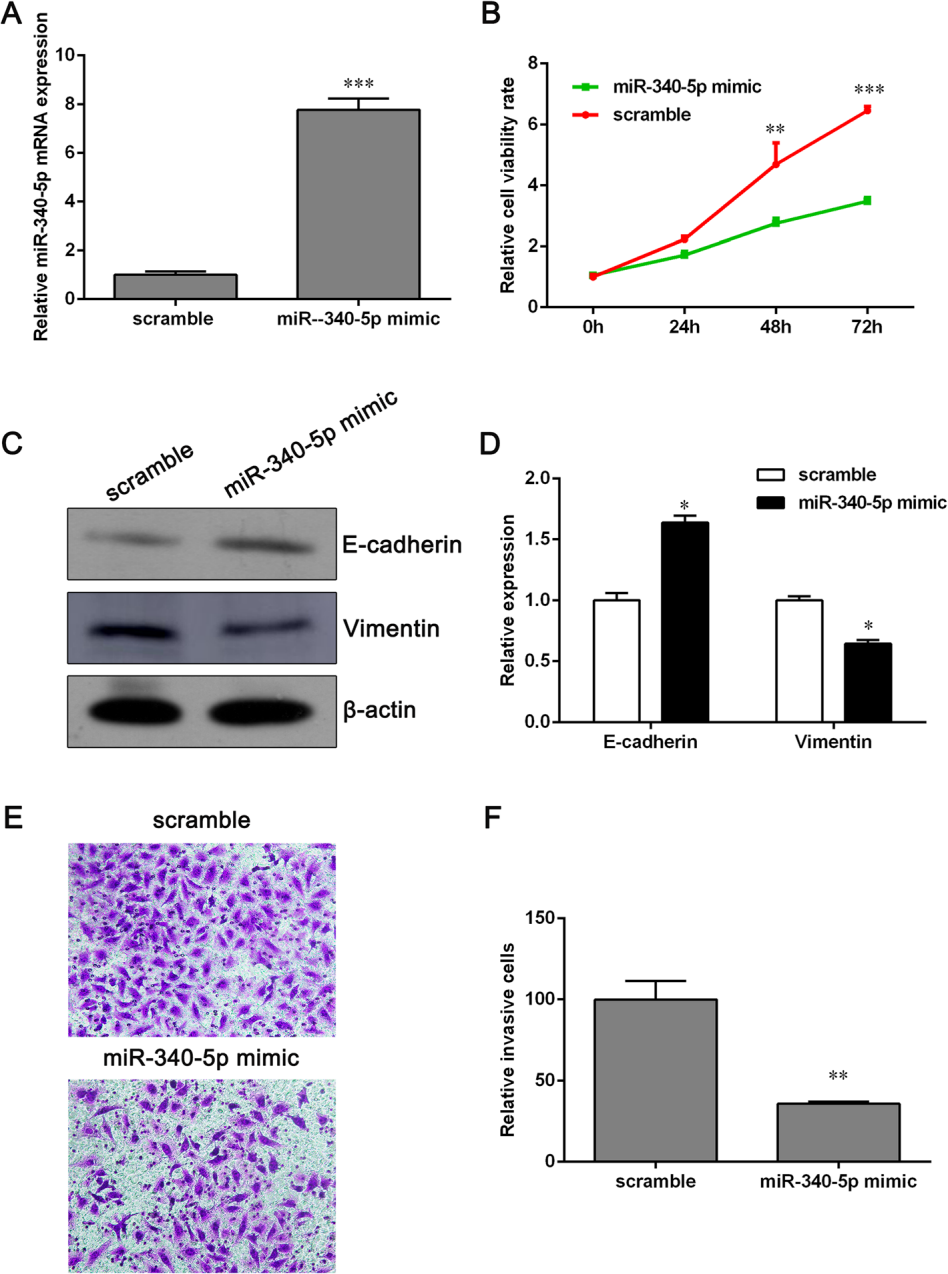
Elevated miR-340-5p inhibits the proliferation and invasion of NCI-H1650 cells. **a** The expression of miR-340-5p in NCI-H1650 cells transfected with miR-340-5p mimics was determined using qPCR. **b** Cell viability was determined using CCK-8. **c** Western blot assays were conducted to detect the expressions of E-cadherin and vimentin. **d** The data represent relative protein expression. **e** Transwell invasion assays were performed to assess the effect of overexpressed miR-340-5p on NCI-H1650 cell invasive ability. **f** The number of invaded cells was analyzed using GraphPad Prism 5.0. Data are presented as the means ± SD (*n* = 3). *p < 0.05, **p < 0.01 and ***p < 0.001 versus the scrambled group

To determine if miR-340-5p has an inhibitory effect on NSCLC, miR-340-5p mimics were transfected into NCI-H1650 cells (Fig. 2a). The results indicated that overexpression of miR-340-5p significantly inhibited NCI-H1650 cell viability at 48 h and 72 h compared to the scramble group (Fig. 2b). Moreover, overexpression of miR-340-5p led to a decrease in the level of vimentin, a mesenchymal marker, and increased the expression of the epithelial marker E-cadherin (Fig. 2c and d). Since epithelial–mesenchymal transition (EMT) is associated with tumor metastasis, we performed a transwell invasion assays to determine if miR-340-5p suppresses the invasion of NCI-H1650 cells. As shown in Fig. 2e and f, the invasion of NCI-H1650 cells was significantly inhibited by miR-340-5p mimics.

To confirm whether miR-340-5p has a necessarily inhibitory influence on NCI-H1650 cell proliferation, an experiment was performed with knockdown of miR-340-5p with miR-340-5p inhibitors. The results show that silencing miR-340-5p promoted cell growth significantly (Additional file 1: Figure S1A and B). These results implied that miR-340-5p may be an important tumor suppressor.

### MiR-340-5p directly targets ZNF503 in NCI-H1650 cells and miR-340-5p expression is negatively correlated with ZNF503 in NSCLC tissues

To determine the mechanism by which miR-340-5p affects the functions of NCI-H1650 cells, we predicted miR-340 targets using TargetScan, focusing on target genes that encode proteins involved in enhancing cancer cell proliferation and invasion. The zinc finger protein ZNF503, which plays a positive role in cancer cell growth and invasion [17], was found to be a novel target candidate of miR-340-5p: the 217–224 position of the ZNF503 3′-UTR was complementary to the seed sequence of miR-340-5p.

**Fig. 3:**
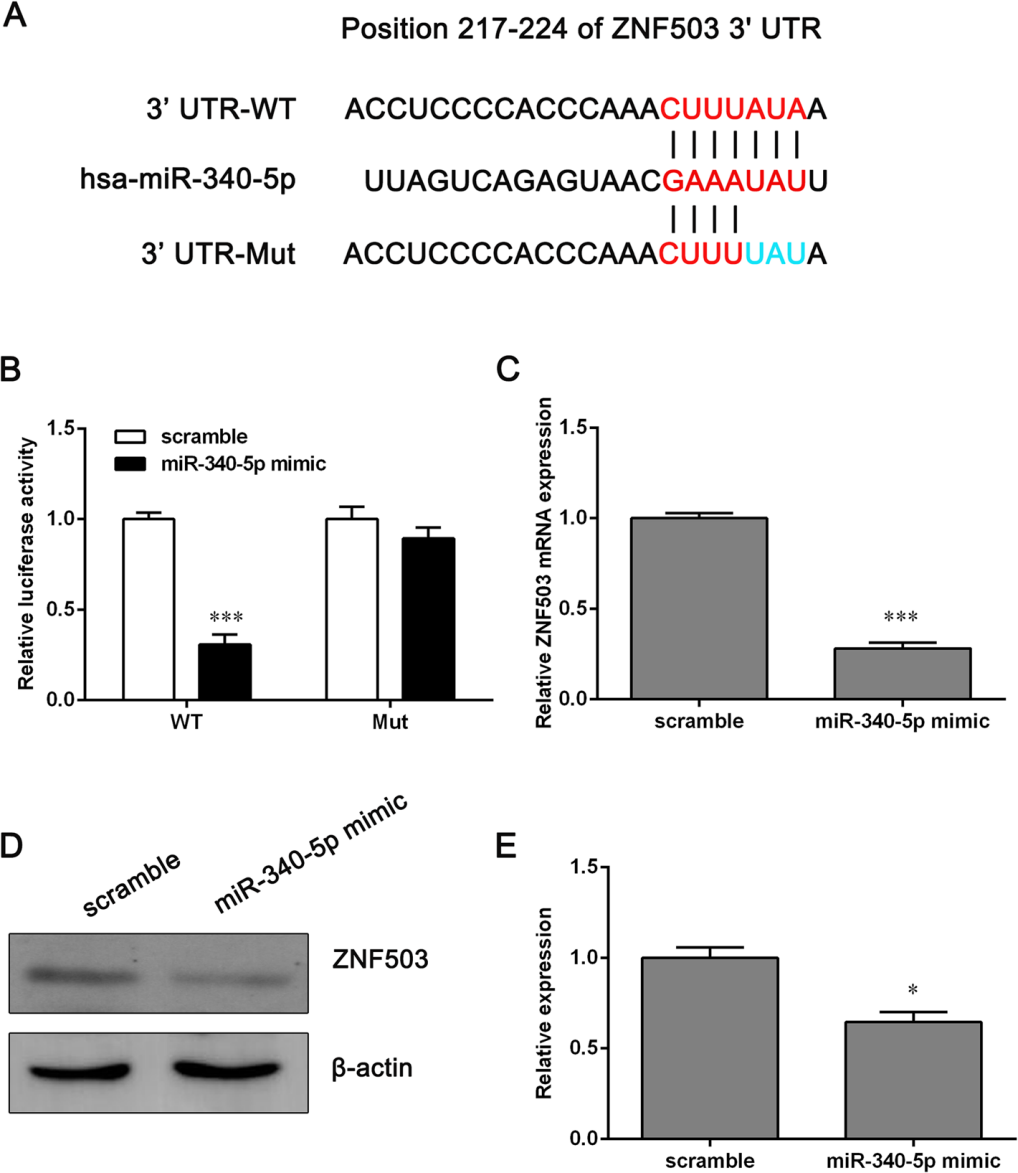
MiR-340-5p directly targets ZNF503 in NCI-H1650 cells. **a** The presumptive binding sites of miR-340-5p within the WT-3′-UTR-ZNF503 and the Mut-3′-UTR-ZNF503 are shown. **b** A dual luciferase reporter assay was used to evaluate the binding specificity between miR-340-5p and WT-3′-UTR-ZNF503. **c** The mRNA level of ZNF503 in NCI-H1650 cells transfected with miR-340-5p mimics was determined using a qPCR assay. **d** The expression of ZNF503 protein was determined via western blotting after NCI-H1650 cells were transfected with miR-340-5p mimics. **e** The relative protein expression is shown. Data are expressed as the means ± SD (*n* = 3). *p < 0.05 and **p < 0.001 versus the scrambled group

To validate this potential direct interaction, luciferase reporter vectors containing the WT and Mut 3′-UTR of ZNF503 were constructed (Fig. 3a). The dual luciferase reporter assays demonstrated that the luciferase activity of WT-3′-UTR-ZNF503 was decreased significantly by overexpression of miR-340-5p, while the luciferase activity of Mut-3′-UTR-ZNF503 remained unchanged (Fig. 3b). Furthermore, we found that the mRNA and protein levels of ZNF503 showed a significant decrease in NCI-H1650 cells that were transfected with miR-340-5p mimics (Fig. 3c, d and e). These findings indicated that miR-340-5p may directly target ZNF503 in NCI-H1650 cells.

We performed qPCR assays to determine the expression levels of ZNF503 in 15 paired NSCLC tissue and pericarcinomatous normal tissue samples and analyzed the correlation between the expression levels of miR-340-5p and ZNF503 in NSCLC tissues. The results indicated that ZNF503 expression was significantly upregulated in NSCLC tissues compared with adjacent non-tumor tissues (Additional file 2: Figure S2A and B). Moreover, we verified that there was a significant inverse correlation between miR-340-5p and ZNF503 expression in the NSCLC tissues (Additional file 2: Figure S2C).

### Overexpressed ZNF503 reverses the inhibition of NCI-H1650 cell proliferation and invasion by miR-340-5p

Based on previous results, we speculated that ZNF503 was a direct target of miR-340-5p. However, whether ZNF503 could rescue the anti-tumor effect of NCI-H1650 cell remains unclear. Therefore, we co-transfected a ZNF503 plasmid and miR-340-5p mimics into NCI-H1650 cells.

**Fig. 4:**
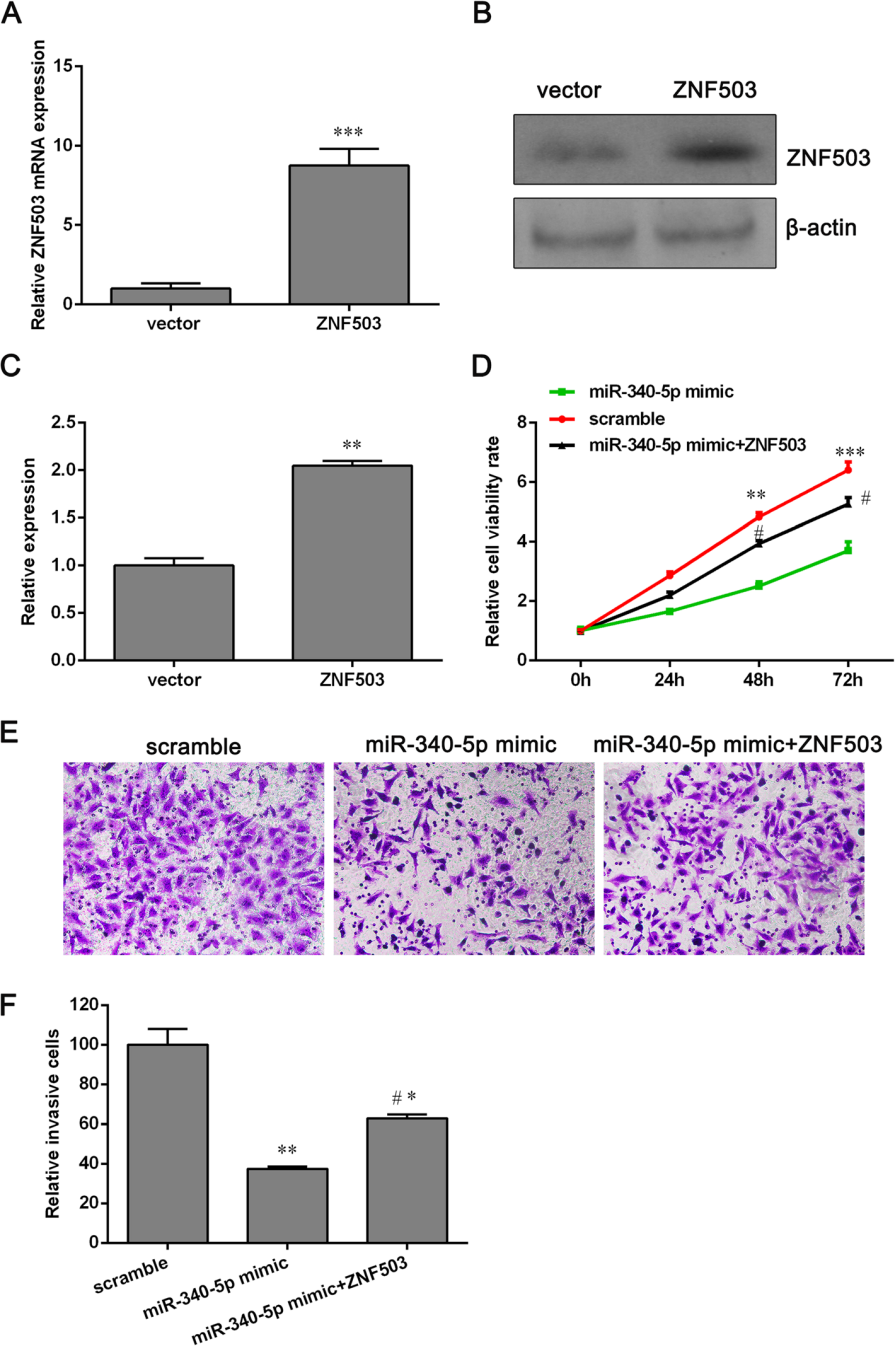
Overexpression of ZNF503 reverses the inhibition of NCI-H1650 cell proliferation and invasion by miR-340-5p. **a** and **b** The mRNA (**a**) and protein (**b**) levels of ZNF503 in NCI-H1650 cells that were transfected with ZNF503 plasmids or vectors were respectively analyzed using qPCR or western blotting. **c** The data demonstrate relative protein expression. **d** The cell viability of miR-340-5p-overexpressing NCI-H1650 cells was partially elevated after overexpression of ZNF503. **e** Ectopic expression of ZNF503 facilitated cell invasion in NCI-H1650 cells that overexpress miR-340-5p. **f** The relative number of invasive cells is shown. Data are presented as the means ± SD (*n* = 3). *p < 0.05, **p < 0.01 and ***p < 0.001 versus the vector group or the scrambled group. ^#^*p* < 0.05 versus the miR-340-5p mimic group

To verify that there was no problem with ZNF503 overexpression plasmids, we conducted qPCR and western blot assays to detect the mRNA and protein expression levels of ZNF503 after transfection. The levels of mRNA and protein expression of ZNF503 were significantly higher after transfection with the ZNF503 plasmid (Fig. 4a, b and c). In addition, the cell viability and invasion of NCI-H1650 cells that were co-transfected with the ZNF503 plasmid and miR-340-5p mimics were higher than those that were co-transfected with miR-340-5p mimics and a mock control of the ZNF503 vector, implying that the overexpression of ZNF503 could reverse the inhibition of NCI-H1650 cell proliferation and invasion caused by miR-340-5p (Fig. 4d, e and f).

We conducted several new experiments to determine if miR-340-5p–ZNF503-mediated regulation of cell proliferation and invasion also occurs in other NSCLC cell lines, such as the A549 cell line. The results Show that overexpression of miR-340-5p markedly decreased the A549 cell proliferation rate compared with the scramble group. However, the cell viability was elevated in A549 cells that were co-transfected with the ZNF503 plasmid and miR-340-5p mimics (Additional file 3: Figure S3A). Transwell invasion assays showed a similar effect. The ectopic expression of miR-340-5p attenuated the A549 cell invasion ability, while the invasion potential of miR-340-5p-overexpressed cells was enhanced after being transfected with the ZNF503 overexpression plasmid (Additional file 3: Figure S3B and C). These results indicated that miR-340-5p–ZNF503-mediated cell proliferation and invasion is not cell-type specific.

## Discussion

Considerable evidence indicates that miR-340 plays a tumor suppressor role in various cancers [12, 14–16, 18–20]. By upregulating miR-340, Kaempferol inhibits A549 cell proliferation, but induces apoptosis and autophagy [21]. In breast cancer, miR-340 suppresses cell migration and invasion via different mechanisms [12, 18]. In prostate cancer, miR-340 was found to inhibit cell proliferation and metastasis by targeting the MDM3–p53 pathway, and to suppress the tumorigenic potential of prostate cancer cells by targeting the high-mobility group nucleosome-binding domain 5 [13, 20].

Recent research has shown that the expression of miR-340 is lower in NSCLC tissues than in paired adjacent noncancerous lung tissues and that low levels of expression of miR-340 indicate poor prognosis for NSCLC. In vitro experiments verified that miR-340 inhibits NSCLC cell growth and colony formation and induces cell cycle arrest by targeting the CDK4 protein [15]. Another study demonstrated that miR-340 suppresses cell viability and induces apoptosis by increasing the expression of p27 in NSCLC cells [16]. Further mechanistic research found that the accumulation of p27 is due to three post-transcriptional regulators (PUM1, PUM2 and SKP2) of p27 that were decreased by miR-340 [16].

In this study, we collected 15 matched NSCLC tissue and normal lung tissue samples and demonstrated that miR-340-5p is expressed at a lower level in the NSCLC tissues. In addition, miR-340-5p is downregulated in NSCLC cell lines. These results are consistent with those from previous studies. An earlier analysis of NSCLC clinical specimens demonstrated an inverse correlation between miR-340 expression and NSCLC progression [16], indicating the potential of miR-340-5p as an oncosuppressor.

Overexpression of miR-340-5preduces NCI-H1650 cell viability and invasion ability. This results from a decrease in the mesenchymal marker vimentin, and an increase in the epithelial marker E-cadherin. However, miR-340-5p inhibitors stimulate cell proliferation. Our observations are similar to those in previous reports.

Next, we verified that miR-340-5p-mediated NCI-H1650 cell growth suppression is associated with the expression of ZNF503, which is inhibited by miR-340-5p. This suggests that the underlying mechanism for the inhibition of miR-340-mediated NSCLC cell proliferation may vary.

Zinc finger proteins, the largest transcription factor family, are crucial for modulating gene expression and are therefore involved in various biological process, including different aspects of tumorigenesis. For instance, ZKSCAN3 (ZNF306) promotes cell growth, migration, angiogenesis and proteolysis in colorectal cancer [22, 23]. ZNF322A promotes cell proliferation, migration and invasion [24]. ZNF503, a transcriptional repressor, was reported to promote mammary epithelial cell growth and to reinforce cell invasion by repressing GATA3 expression and its transcriptional activity. The transcription factor GATA3 is a master regulator that drives mammary luminal epithelial cell differentiation and maintains mammary gland homeostasis [17]. Zheng et al. found that ZNF503 facilitates the proliferation of colon cancer cells and plays an important role in tumor progression by activating the oncogene Myc [25].

Here, we used TargetScan to predict the target of miR-340-5p and found that miR-340-5p directly targets the 3′-UTR of ZNF503. To understand the role that ZNF503 plays in miR-340-5p-mediated cell proliferation and the inhibition of invasion, NCI-H1650 cells were transiently co-transfected with miR-340-5p mimics and ZNF503 overexpression plasmids. The results indicate that the mRNA and protein levels of ZNF503 were downregulated by miR-340-5p, and that overexpression of ZNF503 could antagonize the inhibitory effect on NCI-H1650 cells that was triggered by miR-340-5p (Fig. 4). Since ZNF503 has been reported to increase cell viability and the capacity of invasion [17], our results suggest that the NCI-H1650 cell growth and invasion that was inhibited by miR-340-5p should be dependent on ZNF503 and that inhibition of ZNF503 may synergize with miR-340-5p against NSCLC.

Currently, the underlying molecular mechanisms involved in ZNF503-mediated NSCLC cell proliferation and invasion remain unknown. We intend to conduct additional investigations in this area.

## Conclusion

We found that the expression of miR-340-5p is at a low level in NSCLC tissues and cell lines. This study provides evidence that miR-340-5p inhibits NCI-H1650 cell proliferation and invasion by negatively regulating ZNF503 expression. To the best of our knowledge, ours is the first report of a direct relationship between miR-340-5p and ZNF503. MiR-340-5p may be a potential target for the treatment of NSCLC.

## Additional files

Additional file 1: **Figure S1.** Decreased miR-340-5p promotes proliferation of NCI-H1650 cells. A – The expression of miR-340-5p in NCI-H1650 cells transfected with miR-340-5p inhibitors was measured using qPCR. B – Cell viability was determined using CCK-8. Data are expressed as the means ± SD (*n* = 3). *p < 0.05 and **p < 0.001 versus the scrambled group. (TIF 166 kb)

Additional file 2: **Figure S2.** Upregulation of ZNF503 is observed in NSCLC tissues. A – The mRNA level of ZNF503 in 15 paired NSCLC tissues and adjacent normal tissues was determined with qPCR. B – The expression of ZNF503 is notably higher in NSCLC tissues than that in normal lung tissues. C – The correlation between miR-340-5p and ZNF503 expression levels was analyzed with GraphPad Prism 5.0 software. Data are expressed as the means ± SD. ***p < 0.001 versus the normal tissue group. (TIF 222 kb)

Additional file 3: **Figure S3.** Upregulation of ZNF503 rescues the inhibition of A549 cell proliferation and invasion by miR-340-5p. A – The cell proliferation of miR-340-5p overexpressing A549 cells was partially increased after upregulation of ZNF503. B – Ectopic expression of ZNF503 promoted cell invasion in the A549 cells overexpressing miR-340-5p. C – The relative number of invasive cells is shown. Data are shown as the means ± SD (*n* = 3). *p < 0.05, and ***p < 0.001 versus the scrambled group. ^#^*p* < 0.05 versus the miR-340-5p mimic group. (TIF 3306 kb)

Additional file 4: **Table S1.** Relationship between miR-340-5p expression and the 15 NSCLC patients’ clinical parameters. **Table S2.** Apoptotic rate induced by miR-340-5p overexpression. (DOCX 17 kb)

## Data Availability

The datasets supporting the conclusions of this article are included within the article and its additional files.

## Abbreviations

EMT: Epithelial–mesenchymal transition
miRNAs: MiRNAs MicroRNAs
Mut: Mutant
NSCLC: Non-small cell lung cancer
SCLC: Small cell lung cancer
UTR: Untranslated region
WT: Wild type
