# Supplementary figures and images for: MicroRNA-340-5p suppresses non-small cell lung cancer cell growth and metastasis by targeting ZNF503
Source: Cell Mol Biol Lett. 2019 May 28;24:34. doi: 10.1186/s11658-019-0161-1 (PMC6537386; doi:10.1186/s11658-019-0161-1)

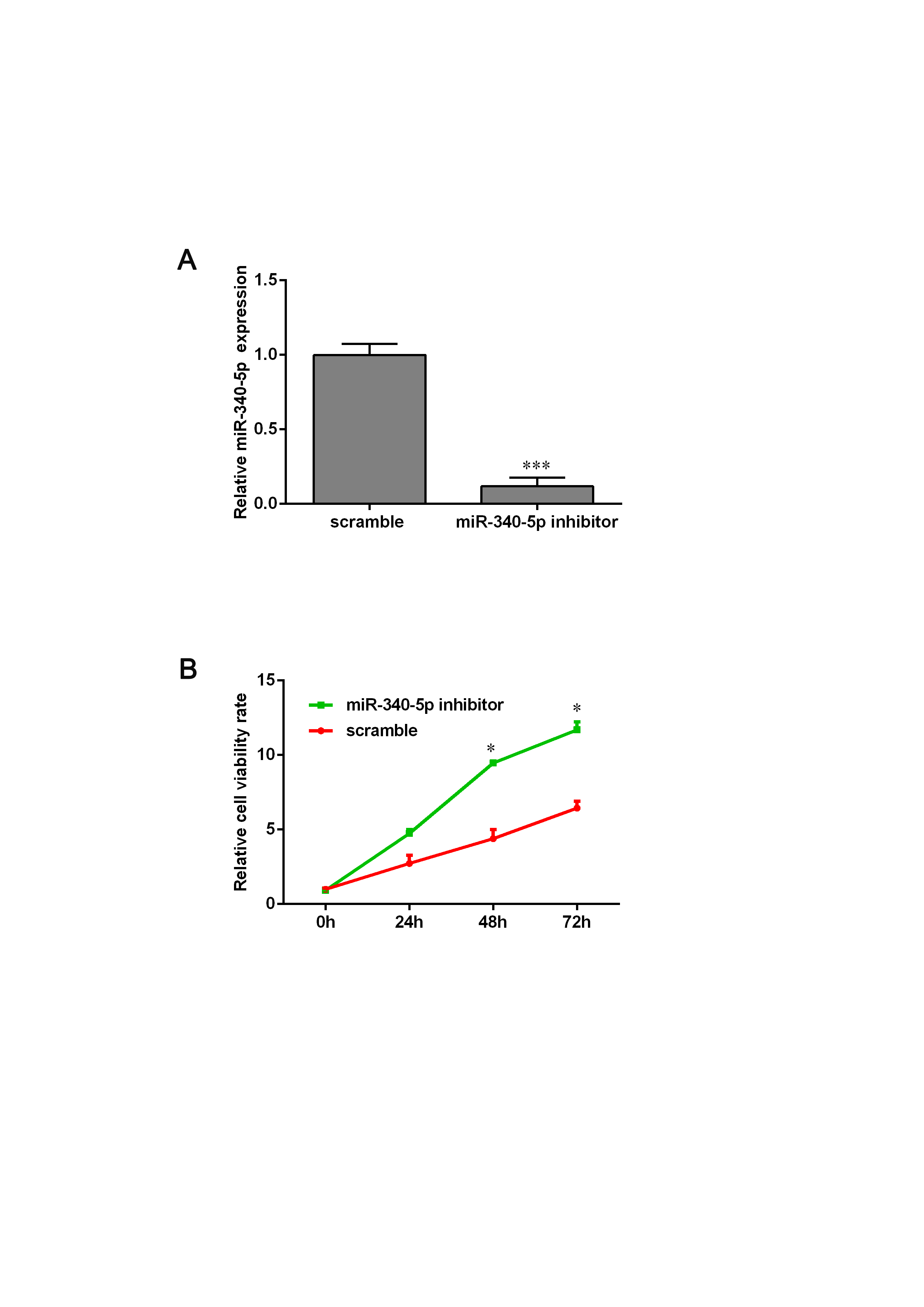

Supplement: Supplementary file 1 — Figure S1. Decreased miR-340-5p promotes proliferation of NCI-H1650 cells. A – The expression of miR-340-5p in NCI-H1650 cells transfected with miR-340-5p inhibitors was measured using qPCR. B – Cell viability was determined using CCK-8. Data are expressed as the means ± SD (n = 3). *p < 0.05 and **p < 0.001 versus the scrambled group. (TIF 166 kb) [file 11658_2019_161_MOESM1_ESM.tif]

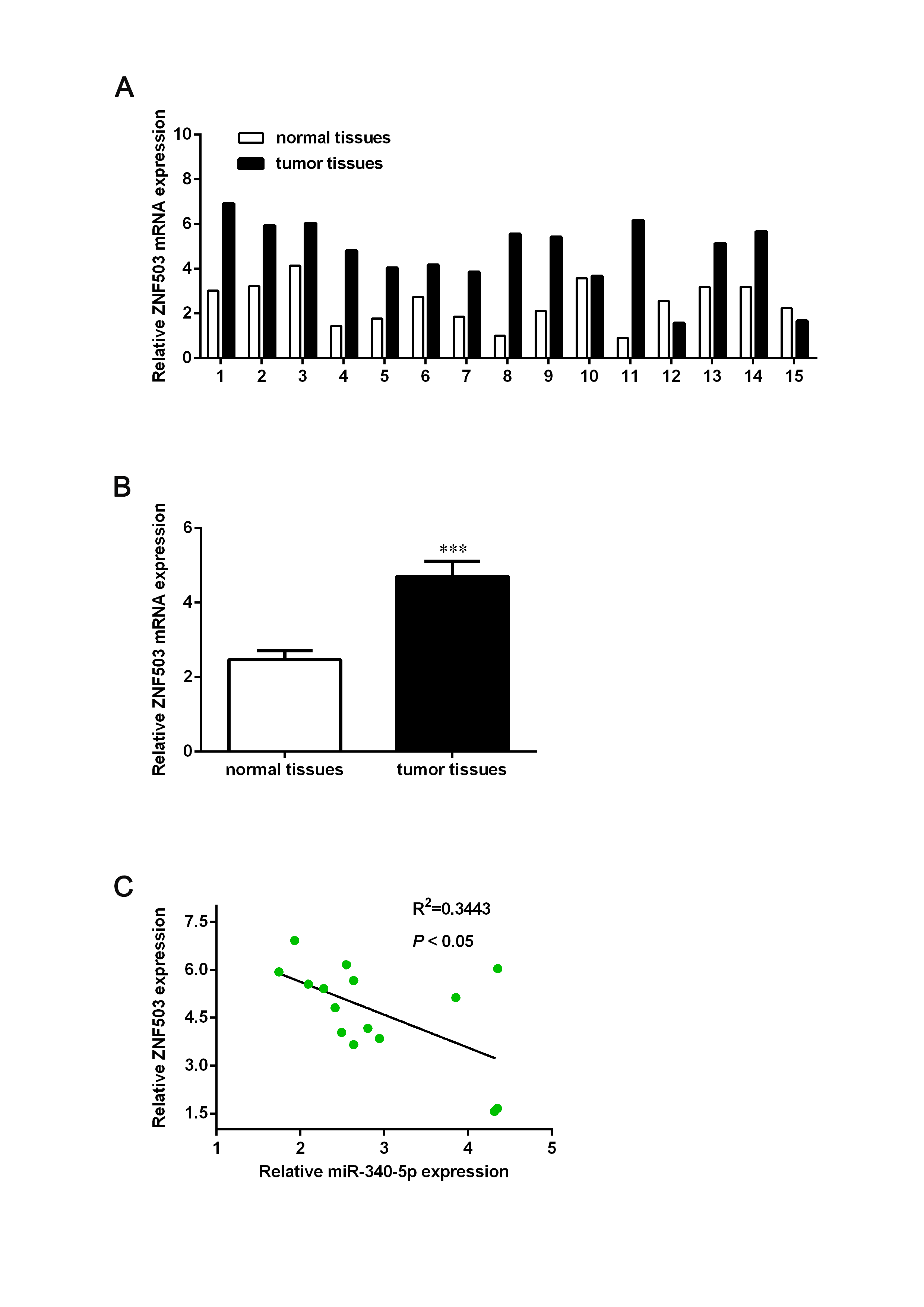

Supplement: Supplementary file 2 — Figure S2. Upregulation of ZNF503 is observed in NSCLC tissues. A – The mRNA level of ZNF503 in 15 paired NSCLC tissues and adjacent normal tissues was determined with qPCR. B – The expression of ZNF503 is notably higher in NSCLC tissues than that in normal lung tissues. C – The correlation between miR-340-5p and ZNF503 expression levels was analyzed with GraphPad Prism 5.0 software. Data are expressed as the means ± SD. ***p < 0.001 versus the normal tissue group. (TIF 222 kb) [file 11658_2019_161_MOESM2_ESM.tif]

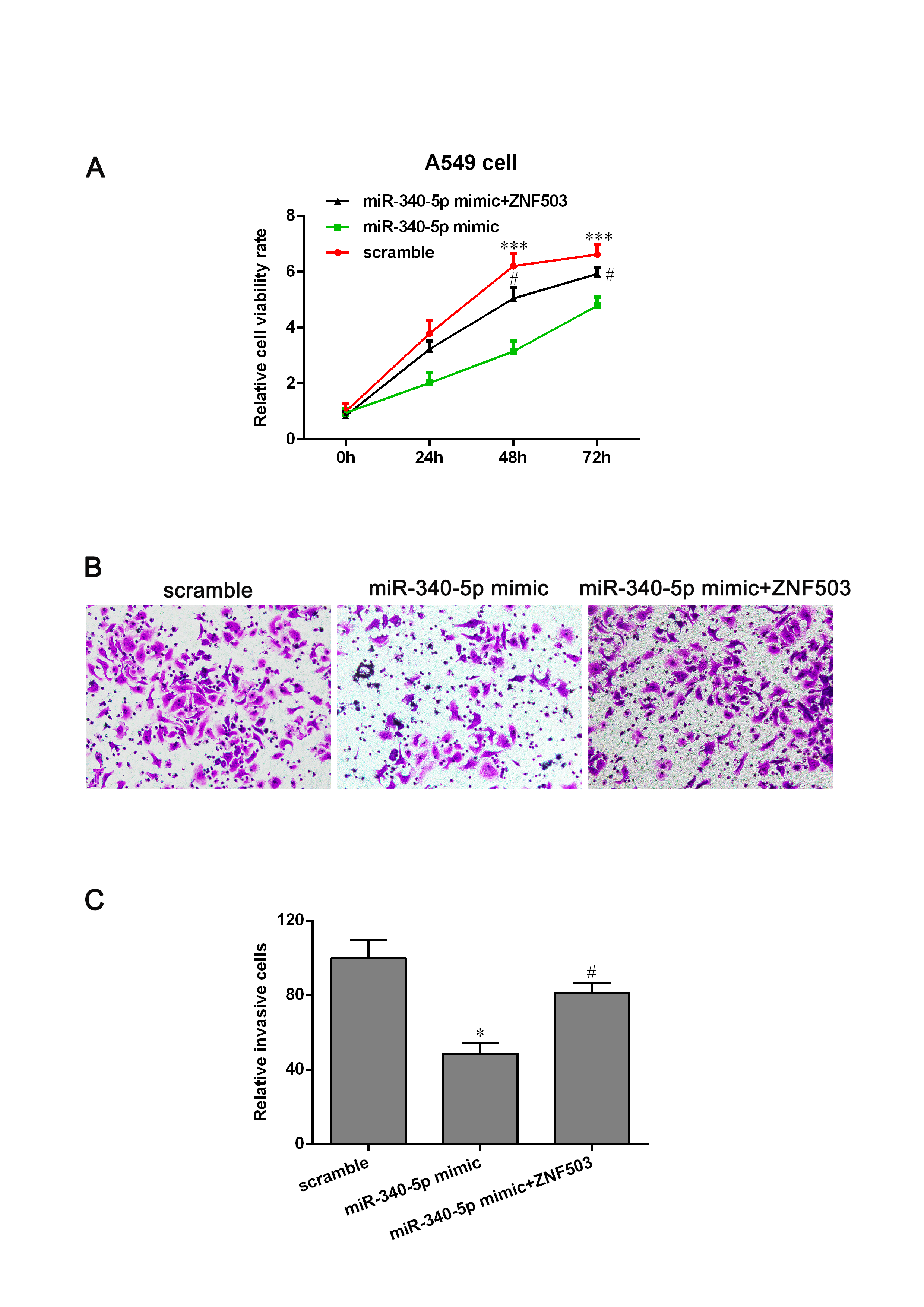

Supplement: Supplementary file 3 — Figure S3. Upregulation of ZNF503 rescues the inhibition of A549 cell proliferation and invasion by miR-340-5p. A – The cell proliferation of miR-340-5p overexpressing A549 cells was partially increased after upregulation of ZNF503. B – Ectopic expression of ZNF503 promoted cell invasion in the A549 cells overexpressing miR-340-5p. C – The relative number of invasive cells is shown. Data are shown as the means ± SD (n = 3). *p < 0.05, and ***p < 0.001 versus the scrambled group. #p < 0.05 versus the miR-340-5p mimic group. (TIF 3306 kb) [file 11658_2019_161_MOESM3_ESM.tif]
