# Supplementary material for: MicroRNA-340-5p suppresses non-small cell lung cancer cell growth and metastasis by targeting ZNF503
Source: Cell Mol Biol Lett. 2019 May 28;24:34. doi: 10.1186/s11658-019-0161-1 (PMC6537386; doi:10.1186/s11658-019-0161-1)
Supplement: Supplementary file 4 — Table S1. Relationship between miR-340-5p expression and the 15 NSCLC patients’ clinical parameters. Table S2. Apoptotic rate induced by miR-340-5p overexpression. (DOCX 17 kb) [file 11658_2019_161_MOESM4_ESM.docx]

**Additional file 4: Table S1.** **Relationship between miR-340-5p expression and**

**the 15 NSCLC patients’ clinical parameters**

| Parameters | MiR-340-5p expression | | *P*-value |
| --- | --- | --- | --- |
|  | High (n=8) | Low (n=7) |  |
| Age |  |  | 0.6193 |
| ≤55 | 3 | 4 |  |
| ＞55 | 5 | 3 |  |
| Sex |  |  | 0.6084 |
| Male | 4 | 5 |  |
| Female | 4 | 2 |  |
| Clinical stage |  |  | 0.0406 |
| Ⅰ or Ⅱ | 7 | 2 |  |
| Ⅲ or Ⅳ | 1 | 5 |  |
| Metastasis |  |  | 0.0406 |
| No | 6 | 1 |  |
| Yes | 2 | 6 |  |

*P*-value was derived by a two-sided Pearson chi-square test.

**Table S2. Apoptotic rate induced by miR-340-5p overexpression**

| MiR-340-5p mimics dosage (nM) | 0 | 2 | 4 | 6 | 8 | 10 |
| --- | --- | --- | --- | --- | --- | --- |
| Apoptotic rate (%) | 4.89 | 4.73 | 5.01 | 11.09 | 20.4 | 25.2 |
